# GABA-A Channel Subunit Expression in Human Glioma Correlates with Tumor Histology and Clinical Outcome

**DOI:** 10.1371/journal.pone.0037041

**Published:** 2012-05-17

**Authors:** Anja Smits, Zhe Jin, Tamador Elsir, Hugo Pedder, Monica Nistér, Irina Alafuzoff, Anna Dimberg, Per-Henrik Edqvist, Fredrik Pontén, Eleonora Aronica, Bryndis Birnir

**Affiliations:** 1 Department of Neuroscience, Neurology, Uppsala University, Uppsala, Sweden; 2 Department of Neuroscience, Molecular Physiology and Neuroscience, Uppsala University, Uppsala, Sweden; 3 Department of Oncology-Pathology, Karolinska Institute, Karolinska University Hospital, Stockholm, Sweden; 4 Department of Immunology, Genetics and Pathology, Uppsala University, Uppsala, Sweden; 5 Department of (Neuro)pathology, Academic Medical Center, Amsterdam, The Netherlands; 6 Stichting Epilepsie Instellingen Nederland, Heemstede, The Netherlands; The University of Chicago, United States of America

## Abstract

GABA (γ-aminobutyric acid) is the main inhibitory neurotransmitter in the CNS and is present in high concentrations in presynaptic terminals of neuronal cells. More recently, GABA has been ascribed a more widespread role in the control of cell proliferation during development where low concentrations of extrasynaptic GABA induce a tonic activation of GABA receptors. The GABA-A receptor consists of a ligand-gated chloride channel, formed by five subunits that are selected from 19 different subunit isoforms. The functional and pharmacological properties of the GABA-A channels are dictated by their subunit composition. Here we used qRT-PCR to compare mRNA levels of all 19 GABA-A channel subunits in samples of human glioma (n = 29) and peri-tumoral tissue (n = 5). All subunits except the ρ1 and ρ3 subunit were consistently detected. Lowest mRNA levels were found in glioblastoma compared to gliomas of lower malignancy, except for the θ subunit. The expression and cellular distribution of the α1, γ1, ρ2 and θ subunit proteins was investigated by immunohistochemistry on tissue microarrays containing 87 gliomas grade II. We found a strong co-expression of ρ2 and θ subunits in both astrocytomas (r = 0.86, p<0.0001) and oligodendroglial tumors (r = 0.66, p<0.0001). Kaplan-Meier analysis and Cox proportional hazards modeling to estimate the impact of GABA-A channel subunit expression on survival identified the ρ2 subunit (p = 0.043) but not the θ subunit (p = 0.64) as an independent predictor of improved survival in astrocytomas, together with established prognostic factors. Our data give support for the presence of distinct GABA-A channel subtypes in gliomas and provide the first link between specific composition of the A-channel and patient survival.

## Introduction

Gliomas are the most common form of primary brain tumor with an overall incidence of about 4–5 per 100.000 persons per year [1], [2]. The majority of gliomas consist of glioblastomas, which are highly proliferative and invasive tumors characterized by remarkable biological heterogeneity and poor response to present treatments [3]. The prognosis for patients with diffuse low-grade gliomas is more favorable, but these tumors transform into malignant gliomas over time with eventually fatal outcome [4]. The etiology of gliomas is largely unknown, with exposure to high-dose ionizing radiation as one of the few recognized risk factors [2].

GABA is the main inhibitory neurotransmitter in the central nervous system. The most common type of GABA receptors is the GABA-A channel, consisting of a ligand-gated pentameric chloride channel that is normally closed but can be opened by GABA [5]. Nineteen different GABA-A channel subunits have been cloned and are grouped into eight separate subfamilies (α1–6, β1–3, γ1–3, δ, ε, θ, π and ρ1–3). All nineteen subunits are expressed in the brain [6]. The GABA-A channel consists most often of three types of subunit isoforms: two αs, two βs and a third type of subunit. All neurons contain GABA-A ion channels but the channel subtypes change during development, varying also between different brain regions and different types of neurons. The distinctive functional and pharmacological of the GABA-A channels are dictated by the composition of the subunits and can be modulated by intracellular proteins [7].

GABA is present in high concentrations in the presynaptic terminals of neuronal cells. In the postsynaptic terminal, brief exposure to high concentration of GABA results in opening of GABA-A channels and a subsequent increase in membrane conductance known as phasic inhibition. It has been shown that GABA-mediated signaling in the brain occurs also in a less spatially and temporally restricted manner, through low concentrations in the extracellular space that result in a persistent or tonic activation [5], [8]. This tonic activation of GABA-A channels was first identified in voltage-clamp recordings in hippocampal and cerebellar neurons but is known to occur more generally in the mammalian brain [9]. Extrasynaptic pharmacologically active GABA-A channels in the mature brain have been found in non-neuronal cells such as astroglial cells [10], [11], and also in T lymphocytes [12], suggesting an immunoregulatory role for extrasynaptic GABA [13].

There is growing evidence also for a widespread role of GABA in the growth regulation of many cell types, including neuronal stem cells and perhaps tumor stem cells [6]. In the postnatal subventricular zone along the lateral ventricle where adult neurogenesis occurs, GABAergic signaling is involved in the proliferative control of neuroblasts [6]. In primary brain tumors, GABA-A channel subunits have been detected in the neuronal component of gangliogliomas [14], but also in gliomas where the response to GABA correlated with the malignancy grade of the tumor [15], [16]. GABA-evoked current response was restricted to low-grade gliomas and not recorded in glioblastoma, suggesting that the disappearance of GABA-A channels parallels the unlimited growth of malignant gliomas [16]. When co-cultured with neurons, an interaction between neurons and glioma cells was triggered resulting in functional expression of GABA-A channels by tumor cells within 24 hours [17].

These reports are all in favor of a role for extrasynaptic GABA in the growth control of gliomas, but so far this has not been substantiated by studies on clinical patient material. Also, a systematical analysis of the distribution of the different GABA-A channel subunit isoforms in human gliomas has not been performed. In the present work we compared mRNA levels of all 19 GABA-A channel subunits in gliomas of different malignancy grade and histological subtype. We then studied the distribution of the α1, γ1, ρ2 and θ subunit proteins *in vivo* in a clinical cohort of diffuse low-grade gliomas and correlated the presence of GABA-A channel subunit proteins with patient survival.

## Results

### Quantification of GABA-A channel subunit mRNAs levels

**Figure 1:**
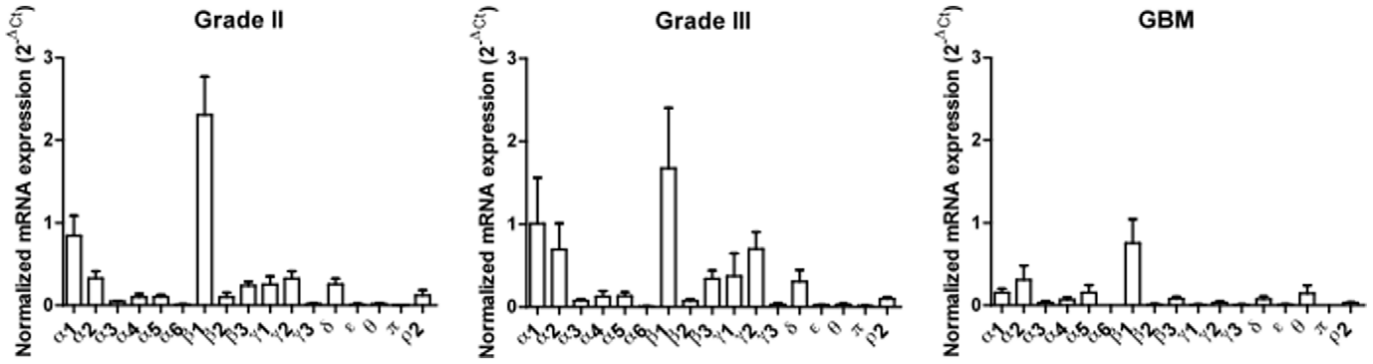
Summary of the qRT-PCR results showing mRNA levels of 17 different GABA-A subunits in gliomas grade II (n = 12), gliomas grade III (n = 10) and glioblastomas (n = 7). The normalized mRNA expression of each target gene relative to a reference gene TATA-binding protein (*TBP*) was calculated using the 2^−ΔCt^ method.

**Figure 2:**
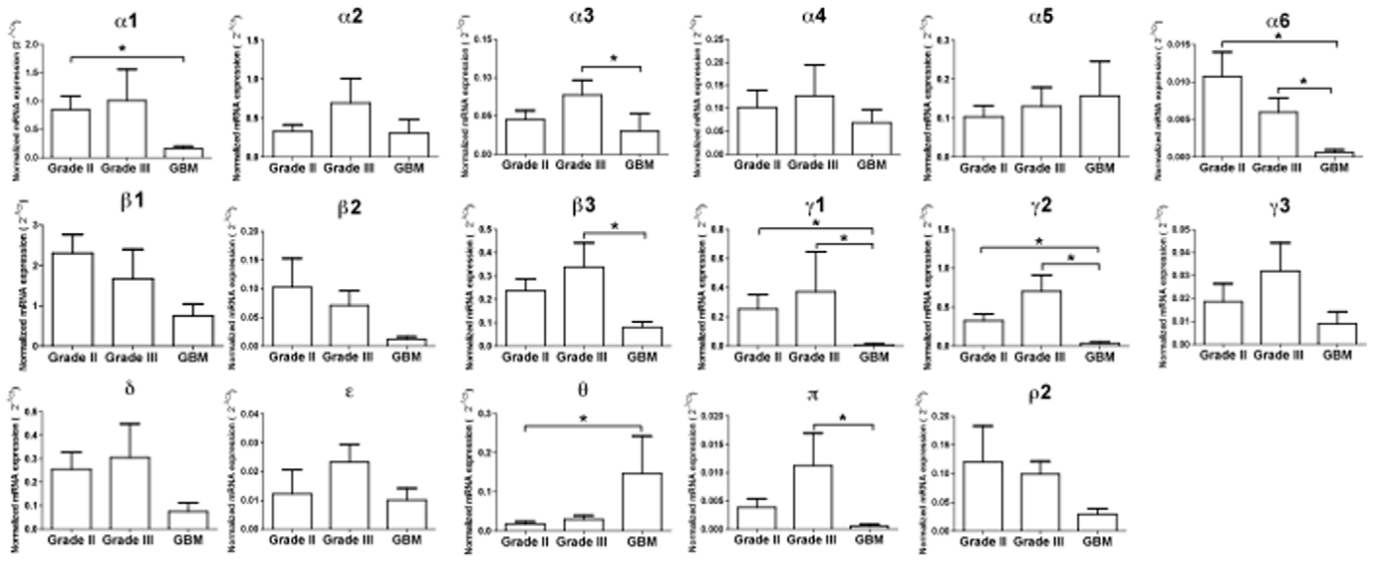
Detailed presentation of the RT-PCR results for each of the 17 subunits, showing quantitative mRNA levels between gliomas grade II, gliomas grade III and glioblastomas. Statistically significant differences in mRNA levels are marked (*). The normalized mRNA expression of each target gene relative to a reference gene TATA-binding protein (*TBP*) was calculated using the 2^−ΔCt^ method.

The histological classification of the tumors samples used for quantitative real-time PCR (qRT-PCR) is shown in Table 1. Mean expression levels of GABA-A channel subunit mRNAs were compared group-wise between gliomas grade II (n = 12), gliomas grade III (n = 10) and glioblastomas (n = 7) according to the World Health Organization (WHO) classification of brain tumors [1]. Seventeen GABA-A channel subunit mRNAs were consistently detected, whereas the ρ1 and ρ3 subunits were not expressed. The relative mRNA levels of the 17 detectable GABA-A subunits in gliomas of different malignancy grade are shown in Figure 1. Statistical analysis of quantitative differences between gliomas grade II, gliomas grade III and glioblastomas showed significantly higher mRNA levels of α1, α6, γ1 and γ2 GABA-A channel subunits in gliomas grade II compared to glioblastomas, and of α3, α6, β3, γ1, γ2 and π subunits in gliomas grade III compared to glioblastomas (p<0.05). In contrast, mRNA levels of the θ subunit were 5–10 fold higher in glioblastomas than in gliomas with lower malignancy grade (p<0.05) (Figure 2).

**Table 1:** Histopathological diagnoses of the samples included in the qRT-PCR (n = 33).

Diagnosis	Number of samples
Gliomas grade II	12
Astrocytomas	6
Oligodendrogliomas	6
Gliomas grade III	10
Astrocytomas	5
Oligodendrogliomas	5
Glioblastomas	7
Peri-tumoral brain	5

For 15 of the 17 detectable GABA-A subunits, no statistically significant differences in mRNA levels were found between astrocytomas and oligodendrogliomas grade II (Figure S1) or between astrocytomas and oligodendrogliomas grade III (Figure S2). The two exceptions were the β1 subunit, for which expression was lower in astrocytomas grade II than in oligodendrogliomas grade II (Figure S1), and the α3 subunit, showing lower expression in astrocytomas grade III than in oligodendrogliomas grade III (Figure S2) (Mann-Whitney Rank Sum Test).

In peri-tumoral brain tissue, mRNAs for the 17 GABA-A channel subunits were consistently detected (Figure S3). For most subunits belonging to the α, β and γ subfamilies, as well as for the δ subunit, mRNA levels were 3–5 fold higher in peri-tumoral brain tissue compared to tumor samples. Other subunits were expressed at approximately similar levels as those found in tumors (Figure S3).

### Distribution of GABA-A channel subunit proteins in gliomas

**Figure 3:**
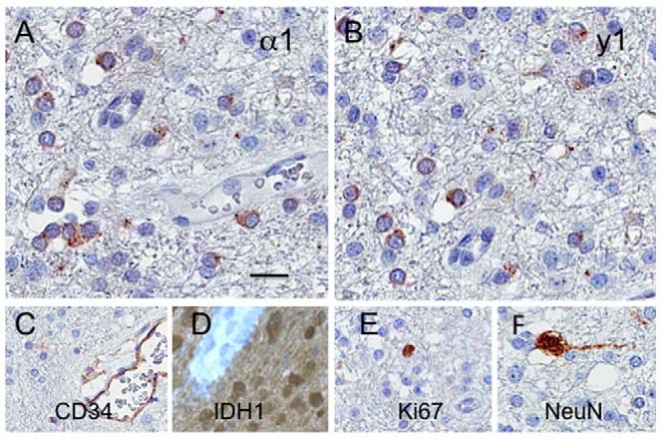
Photomicrographs of an oligodendroglioma grade II showing positive immunoreactivity for: A) GABA-A channel α1 subunit, and B) γ1 subunit. The histological markers used for identification of specific cell types, CD34, IDH1, Ki67 and neuronal nuclear antigen (NeuN) illustrate respectively, C) haematopoetic cells in the tumor, D) accumulation of mutated IDH1 protein in tumor cells, E) a low percentage of proliferating tumor cells, F) an entrapped neuron in the tumor. (The scale bar shown in A represents 50 µm in A, B, F; 80 µm in C, D, E).

Table 2 summarizes patient characteristics and tumor characteristics of the 91 gliomas that were used for immunohistochemical analysis. The results of the immunostainings are shown in Table 3. Tumor cells with immunoreactivity for α1, γ1, ρ2 and θ GABA-A channel subunits were identified in all histological tumor types. The subcellular distribution of the subunit proteins was found to be cytoplasmic and membranous. Of the total number of 91 samples, 17 showed immunoreactivity for the α1 subunit (19%), 24 for the γ1 subunit (26%), 29 for the ρ2 subunit (32%), and 35 for the θ subunit (38%) (Figure 3). The mean fraction of immunoreactive tumor cells denoted as + was between 4–8% for the α1, γ1, ρ2 and θ subunits (Table 3). The mean fraction of immunoreactive tumor cells in astrocytomas denoted as ++ was 15% for the α1 subunit (n = 1), 20% for the γ1 subunit (n = 2), 55% for the ρ2 subunit (n = 5) and 40% for the θ subunit (n = 9). In oligodendrogliomas, the mean fraction of immunoreactive tumor cells denoted as ++ was 30% for the ρ2 subunit (n = 1) and 50% for the θ subunit (n = 2), while in oligoastrocytomas these percentages were 50% for the γ1 subunit (n = 3), 35% for the ρ2 subunit (n = 2), and 40% for the θ subunit (n = 3).

**Table 2:** Clinical and tumor characteristics of gliomas included in the TMA (n = 91).

Parameter	Number of patients (%)
Gender	
Male	56 (62)
Female	35 (38)
Mean age (at onset)	40.3 y
Presenting symptoms	
Seizures	76 (84)
Others	15 (16)
Surgery	
Biopsy only	15 (16)
Subtotal resection	50 (55)
Gross total resection	26 (29)
Tumor location	
Frontal	50 (55)
Fronto-temporal/fronto-parietal	11 (12)
Temporal	15 (17)
Temporo-parietal/parieto-occipital	4 (4)
Central	2 (2)
Multilobular	9 (10)

Histology	Total	LOH 1p/19q	Mutated IDH1 protein
Astrocytoma grade II	42	2	32
Oligoastrocytoma grade II	18	3	17
Oligodendroglioma grade II	27	20	24
Astrocytoma grade III	1	0	0
Oligoastrocytoma grade III	3	1	3

**Table 3:** Results of immunostaining for GABA-A channel subunits on gliomas (n = 91).

Subunit/Histology	Nr of samples (n)	α1	γ1	ρ2	θ	Nr of positive tumor cells*
Astrocytoma grade II	30	23	19	22	21	0
		6	9	7	6	+
		1	2	1	3	++
Gemistocytic astrocytoma	12	11	12	2	2	0
		1	0	6	4	+
		0	0	4	6	++
Oligoastrocytoma grade II	15	13	8	14	12	0
		2	4	1	3	+
		0	3	0	0	++
Minigemistocytic oligoastrocytoma	3	2	3	0	0	0
		1	0	1	0	+
		0	0	2	3	++
Oligodendroglioma grade II	27	23	21	22	18	0
		4	6	4	7	+
		0	0	1	2	++
Astrocytoma grade III	1	0	1	0	0	0
		1	0	1	1	+
		0	0	0	0	++
Oligoastrocytoma grade III	3	2	3	2	2	0
		1	0	1	1	+
		0	0	0	0	++

* 0 = No immunoreactive tumor cells; + = <10% immunoreactive tumor cells; ++ = ≥10% immunoreactive tumor cells.

**Figure 4:**
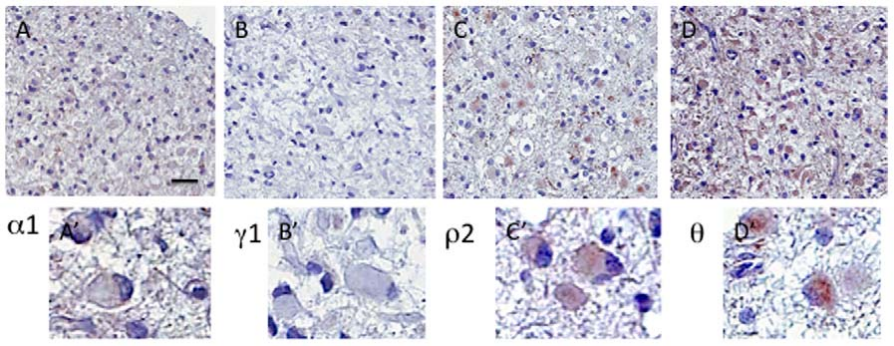
Photomicrographs of a gemistocytic astrocytoma grade II showing no immunoreactivity for: A) and A′) GABA-A channel α1 subunits, B) and B′) γ1 subunits, but positive immunoreactivity for: C) and C′) ρ2 subunits and D) and D′) θ subunits in a proportion of the tumor cells. (The scale bar shown in A represents respectively 200 µm in A, B, C, D; 30 µm in A′, B′, C′, D′).

Three astrocytomas grade II showed immunoreactivity for all four subunits, while none of the oligodendrogliomas and oligoastrocytomas expressed all four subunits. Interestingly, more than 80% of the gemistocytic astrocytomas expressed the ρ2 and θ subunits, but lacked immunoreactivity for the α1 and γ1 subunit proteins (Figure 4). A similar pattern of predominantly ρ2 and θ subunit expression was found in minigemistocytic oligoastrocytomas (Table 3).

### Statistical analysis

**Figure 5:**
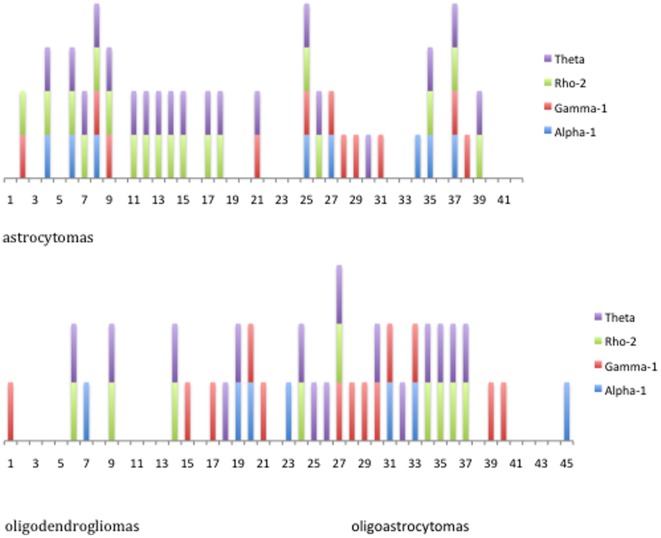
Illustration of the immunohistochemistry results in astrocytomas grade II (n = 42), and in oligodendrogliomas (n = 27) plus oligoastrocytomas grade II (n = 18). Positive immunoreactivity for each of the four different GABA-A channel subunits is visualized by a colored bar. Each number represents one tumor sample.

As a first step, we dichotomized the immunohistochemistry results of the four GABA-A channel subunits into two categories; no immunoreactivity ( = 0) versus positive immunoreactivity (+ or ++). These data are illustrated in Figure 5, where each number in the figure represents one tumor sample consisting of astrocytomas (n = 42), oligodendrogliomas (n = 27) or oligoastrocytomas (n = 18) grade II, with either no (no bar) or positive (colored bar) immunoreactivity for the four subunits. As demonstrated, there was a frequent co-expression of ρ2 and θ subunits in astrocytomas grade II (r = 0.86, p<0.0001) and in oligodendrodendrogliomas/oligoastrocytomas grade II (r = 0.66, p<0.0001). No statistically significant correlation between the α1 and γ1 subunit or between any of the other four subunits was found.

### Survival analysis

The mean postoperative survival for patients with gliomas grade II (n = 87) was 7,9 y. As expected, mean survival was longer amongst patients with oligodendrocytomas compared to oligoastrocytomas and astrocytomas (11.6 y, 6.9 y and 5.9 y respectively). We hypothesized that ρ2 expression and θ subunit expression in grade II gliomas were prognostic factors for survival and tested these two parameter as dichotomized variables in the survival analysis for astrocytomas grade II (n = 42) and for the combined group of oligodendrogliomas and oligoastrocytomas (n = 45).

Both ρ2 subunit expression and θ subunit expression were associated with statistically significant longer median survival in the histological subgroup of astrocytomas (Table 4). Of the established clinical and radiological prognostic factors for grade gliomas grade II (age, epilepsy, KPS, tumor size, tumor location, contrast enhancement) [18], the parameters seizures at onset, KPS≥90 and tumor diameter ≤6 cm were identified as prognostic factors in the univariate analysis (Table 4). Young age (≤40 years), non-enhancing tumor and non-central tumor localization were not associated with favorable prognosis. The presence of mutated IDH1 R132H protein showed a trend towards longer survival in the univariate analysis and was entered as a variable in the multivariate model [19]. Since all patients but one were treated by radiotherapy, this parameter was not tested. Likewise, the LOH 1p/19q status was positive for only two patients and could not be entered into the model. As shown in Table 4, expression of ρ2 subunits contributed to significantly longer survival in the multivariate model, together with seizures at onset, KPS≥90, and mutated IDH1 protein. Expression of θ subunits was not identified as an independent prognostic factor in the multivariate model. Small tumor size was a significantly favorable prognostic factor entered as a single variable (p = 0.019), but lost its significance in the multivariate model.

**Table 4:** Prognostic factors for survival in astrocytomas grade II (n = 42) and oligodendrogliomas/oligoastrocytomas (n = 45) using Log-Rank model (univariate) respectively Cox's proportional hazard model (multivariate).

			Univariate		Multivariate		
Parameter	Nr	median survival	95% CI	p-value	RR	95% CI	p-value
ASTROCYTOMAS	42	4.4	3.7–6.5				
dead/alive	41/1						
*Seizure at onset*:							
no	7	2.0	0.5–2.3	0.0003	12.7	3.5–45.6	0.0002
yes	35	4.8	4.1–7.5				
*Age at onset*:							
<40 y	15	4.3	1.1–8.2	0.73			
≤40 y	27	4.7	3.4–7.2				
*KPS*:							
≥90	26	7.5	3.9–8.6	0.054	0.3	0.2–0.8	0.009
<90	16	4.1	1.5–4.6				
*Tumor size*:							
≥6 cm	14	3.4	1.5–5.7	0.0193	1.1	0.5–2.8	0.89
<6 cm	28	5.3	4.1–8.5				
*Mutation IDH1*:							
yes	32	5.6	3.7–7.4	0.094	0.29	0.1–0.8	0.020
no	10	3.9	1.2–7.9				
*Expression rho2*:							
yes	18	6.5	3.9–8.6	0.029	0.13	0.03–0.9	0.043
no	24	4.1	2.7–5.7				
*Expression theta*:							
yes	19	6.5	4.0–8.6	0.023	1.5	0.2–5.5	0.64
no	23	4.0	2.7–4.9				
OLIGO/O-ASTRO	45	8.3	5.6–9.6				
dead/alive	38/7						
*Histology*:							
oligodendro	27	9.2	4.7–11.3	0.050	0.7	0.5–1.0	0.04
oligoastro	18	7.2	3.1–8.3				
*LOH 1p/19q*:							
no	22	5.6	3.1–9.6	0.22			
yes	23	9.1	7.5–11.3				
*Crossing midline*:							
yes	8	4.5	2.2–8.4	0.002	4.3	1.7–10.1	0.003
no	37	9.2	6.4–11.1				
*Expression rho*2:							
yes	9	6.4	0.3–11.1	0.72			
no	36	8.4	4.7–9.6				
*Expression theta*:							
yes	15	8.1	4.3–9.2	0.30			
no	20	8.3	4.5–11.2				

In the group of oligodendrogliomas and oligoastrocytomas (n = 45), the parameters oligoastrocytoma histology (versus oligodendroglioma) and tumors crossing midline structures (versus non-central tumor location) were identified as unfavorable prognostic factors in both the univariate and multivariate model (Table 4). The parameters expression of ρ2 subunit and expression of θ subunit were not associated with significantly longer survival in these tumors.

## Discussion

The aims of the present study were 1) to explore the distribution of GABA-A channel subunits in human gliomas of various histological subtypes, and 2) to search for correlations between GABA-A channel subunits and patient survival. We showed that 17 of the 19 different GABA-A channel subunit mRNAs were present, only ρ1 and ρ3 subunits could not be detected. While no major differences in subunit mRNA levels were found between astrocytomas and oligodendrogliomas, there was a general down-regulation of GABA-A channel subunits in glioblastoma. Thus, highest mRNA levels were recorded in gliomas grade II and grade III, except for the θ subunit that showed 5–10 fold higher levels in glioblastomas.

Our findings are in agreement with previous reports demonstrating functional bindings sites for the GABA-A channel receptor in low-grade and anaplastic gliomas but not in glioblastomas [16], [17]. The lack of responsiveness to GABA in glioblastomas has been explained by either the loss of GABA-A channels or by defective channels [16]. Thus, tumor cells equipped with functional GABA-A channels may be able to maintain low or intermediate proliferative activity upon physiological levels of GABA in the extracellular fluid. GABA triggered a depolarization in the majority of glioma cells and a concomitant increase of intracellular Ca^2+^ [16]. Since the GABA-A channel itself is not permeable for Ca^2+^ ions, the Ca^2+^ influx has been associated with an activation of voltage-gated Ca^2+^ channels in the tumor cell membrane. The central role of intracellular Ca^2+^ as a downstream effector of GABA receptor signaling in cell proliferation has been confirmed [20].

The other main finding of this study is the strong co-expression of ρ2 and θ subunit proteins in gliomas grade II and the association between ρ2 subunit expression and longer patient survival in diffuse astrocytomas. In spite of its strong co-expression with the ρ2 subunit, the θ subunit did not emerge as an independent predictor of survival in the multivariate model. These findings are in agreement with the qRT-PCR results, showing an opposite trend for the θ subunit with up-regulation in glioblastomas. The prognostic impact of ρ2 subunits in diffuse astrocytomas is intriguing but is not necessarily unique for the GABA A-channel subunit families. It is possible that we failed to identify associations for other subunits in oligodendrogliomas and oligoastrocytomas, which were combined into one group in the present study due to the limited number of tumor samples. Neither can we exclude that there has been a selection bias influencing our results, since many tumor biopsies from the original clinical cohort were too small to be included in the TMA. Thus, our study provides a first link between GABA-A channel composition and survival in gliomas and supports a presumed functional role of GABA in gliomas, but the findings need to be confirmed in larger studies.

The GABA-A channel subunits exist as a family of subtypes with distinct temporal and spatial patterns of expression and distinct properties that presumably underlie a precise role for each subtype [21], [9]. The differential sensitivity to GABA is dependent on the specific subunit composition of the A-channel, and may further be influenced by intracellular proteins interacting with the channel complex and by post-translational modifications [22]. Whilst the α1 subunit has frequently been the focus of studies, due to its abundance in the brain and its reliability of measurement, considerably less is known on the other subunits expressed at a lower level such as θ and the ρ2 subunits. In the brain, functional GABA-A channel receptors containing the θ subunit are formed together with α, β and γ subunits [7]. Synaptic channels are activated by high GABA concentrations, whereas extrasynaptic channels can be activated by extremely low concentrations of GABA. Tumors such as gliomas are probably not exposed to high GABA concentrations and one would expect mostly tonic currents to be active in these tumors. It seems that tonic currents more or less can contain any subunit in their channel complex, whereas synaptic channels are more restricted to certain types of subunits. As such, the γ2 subunit is a major component of synaptic channels, while the α4 α5 α6 and δ subunits are part of extrasynaptic GABA A-channels and thus involved in tonic currents [7], [23]. The α1, α2 and α3 subunits can be located both at and outside of synapses and the ε and θ subunits are probably mostly extrasynaptic [7], [23].

In the present study, ρ2 subunit expression was found in a relatively large number of glioma samples while no detectable mRNA levels were found by qRT-PCR for the ρ1 and ρ3 subunits. The ρ1 subunit is predominantly expressed in the retina and visual pathways, while ρ2 expression has also been found in hippocampus and amygdala [24], [25]. Rho subunits form functional homomeric GABA-A-ρ receptors (previously known as GABA_C_ channels) [7], [26], but can potentially also participate in heteromeric GABA-A channel formation [7], [27]. The GABA-A-ρ receptor has a number of distinctive and unique functional properties, such as a long mean opening time of the channel and slow desensitization, suggesting a more widespread function than previously thought [28]. In addition, the pharmacological properties of the GABA-A-ρ receptor, including the lack of response to benzodiazepines and barbiturates, set apart this class of receptor.

We found a strong positive correlation between the expression of θ and ρ2 subunits. Expression of the θ and ρ2 subunits in brain tissue is relatively uncommon, and there is no evidence to suggest that they form a functional channel together [29], [30]. However, normal transcriptional regulation in tumor cells is disrupted and may not follow the normal “rules” for GABA-A channel subunit expression. It is therefore possible that they could have been expressed without participating in forming functional channels. Interestingly, the strongest correlation between the expression of ρ2 and θ subunits was found in gemistocytic astrocytomas. The presence of gemistocytes in a tumor has been correlated with worse prognosis [31], [32], although the proportion of gemistocytes in the tumor does not seem to have an effect [32]. The reasons for development of gemistocytes in tumors are still poorly understood, but it has been suggested that they may develop due to losing the competition for substrates with adjacent cells [31], [32].

It is not known whether tumor cells are the source of GABA. Only neurons contain glutamic acid decarboxylase (GAD), the enzyme that catalyzes the decarboxylation of glutamate to GABA, and are able to produce GABA. However, astrocytes can take up GABA and there is a minor pathway not involving GAD that allows synthesis of GABA [33]. Both neurons and astrocytes can release GABA, but only neurons can release high concentrations of GABA within a short time-span from synaptic vesicles at the presynaptic terminal [33].

### Conclusions

The present study shows a down-regulation of subunit mRNA levels in glioblastomas, except for the θ subunit, and the presence of distinct GABA-A channel subunit proteins in gliomas grade II. The correlation between ρ2 subunits and favorable survival in diffuse astrocytomas suggests that specific subunit compositions affect clinical outcome in glioma. Taken the distinct pharmacological properties of the different GABA-A channel subtypes, this may open up for new therapeutic strategies for glioma. Our study also highlights the complexity of GABA signaling in gliomas and stresses the need for functional studies in this field.

## Materials and Methods

### Ethics Statement

Informed consent was obtained for the use of human brain tissue and for access to medical records for research purposes, and all material was obtained in a manner compliant with the Declaration of Helsinki. The studies involving human tissue samples were approved by the Ethics Committee of Uppsala University (Application Dnr Ups 02-330) and the Ethics Committee of Karolinska Institutet (Application Dnr Ki 02-254). Written informed consent was obtained prior to sample collection.

### Tissue Samples for quantitative real-time quantitative PCR

Human tissue samples for quantitative real-time quantitative PCR (qRT-PCR) were obtained from the files of the department of neuropathology of the Academic Medical Center, University of Amsterdam. Histological classification of tumors according to the WHO is shown in Table 1. Twenty-nine gliomas (12 gliomas grade II, 10 gliomas grade III, 7 primary glioblastomas), all with temporal lobe location, were included. Five samples of peri-tumoral brain derived from patients with primary glioblastomas in the temporal lobe were included. These samples consisted of tumor-free cortex on the basis of absence of immunoreactivity for Ki67 (1∶500; Rabbit polyclonal, DAKO), IDH1 R132H (1∶100; Clone H09, Dianova) and p53 (1∶2000; Clone DO-7+BP53-12, Neomarkers). Samples were quickly removed at surgery and immediately divided into two parts; one part was fixed in 4% paraformaldehyde for 24 hours, paraffin embedded and used for histopathological diagnosis, the remaining part was snap frozen in liquid nitrogen and maintained at −80°C until used for RNA isolation.

### Quantitative real-time PCR

Total RNA was isolated with TRIzol reagent according to the manufacture's instruction and further transcribed to cDNA using oligodT primers (Table S1). qRT-PCR was performed in a 10 µl reaction mixture containing 4 µl cDNA (0.5 ng/µl), 1× PCR reaction buffer, 3 mM MgCl_2_, 0.3 mM dNTP, 1× ROX reference dye, 0.8 U JumpStart *Taq* DNA polymerase (Sigma-Aldrich), 5× SYBR Green I (Invitrogen) and 0.4 µM each of forward and revere primers. The gene-specific primer pairs were designed using Primer Express Software version. 3.0 (Applied Biosystems), synthesized by Invitrogen and further validated using BioBank cDNA from human brain (PrimerDesign). The specific primer sequences for the 19 different GABA-A subunits are shown in the supplementary material (Table S1). Amplification was performed in 96-well optical plates using the ABI PRISM 7900HT Sequence Detection System (Applied Biosystems) with an initial denaturation of 5 min at 95°C, followed by 45 cycles of 95°C for 15 s, 60°C for 30 s and 72°C for 30 s. A melting curve was determined at the end of cycling to ensure the amplification of a single PCR product. Each reaction was run in duplicate. Cycle threshold values (Ct) were determined with the SDS 2.3 software supplied with the instrument. The expression of each target gene relative to a reference gene TATA-binding protein (*TBP*) was calculated using the 2^−ΔCt^ method as previously described [34].

### Tissue samples for immunohistochemistry

The distribution of GABA-A channel subunit proteins was explored by immunohistochemistry in a tissue microarray (TMA) of glioma samples. The TMA was prepared from paraffin-embedded tissue blocks as previously described [35]. Samples were included from patients aged ≥16 years old operated for low-grade gliomas between 1984–2001 at the Uppsala University Hospital, as part of a previously described clinical cohort of diffuse low-grade gliomas [36]. Paraffin-embedded tissue blocks were identified and re-used for preparation of the TMA. All tumors included in the TMA were re-evaluated for the purpose of the study by one of the neuropathologists (IA). Of the original cohort of 116 grade II gliomas, 3 tumors were re-classified as non-gliomas, 18 blocks contained too little tumor for TMA preparation (mainly biopsies), and in 4 blocks areas with infiltrative tumor edge were left without representative areas of tumor bulk. Thus, 91 glioma samples remained and were included in the TMA presented in this study. Clinical and histopathological tumor characteristics are presented in Table 3.

### Clinical data collection

A retrospective chart review was performed for all tumor cases as previously described [36]. The following clinical data were collected: time-point of first symptoms, patient age at disease onset, date of operation, date of death, tumor size, tumor location (specific lobe, and (sub)cortical versus central location), date and extent of diagnostic surgery (biopsy, subtotal or gross total resection). The extent of tumor resection was based on postoperative CT scans or on the surgeon's operative notes. Survival was defined as the time point between operation and date of death or end of the study (20 September 2009). Data concerning time of death and the cause of death were collected from central health authorities (the National Cause of Death Register Data).

### Tissue microarray preparation

TMAs were prepared by the Swedish Proteome Resource Centre (HPR) facilities at the department of pathology, the Rudbeck laboratory, Uppsala University Hospital [37]. In brief, donor blocks were sectioned, hematoxylin-eosin stained and immunostained with immunohistochemical markers to select appropriate areas for the TMA construct. Doublets of representative areas were included for each tumor. Using an automated tissue arrayer (Beecher Instruments, Silver Spring, MD), two 1.0-mm diameter punches were taken from each donor block and transferred to the recipient TMA block. In total, two TMA blocks were prepared.

### Immunohistochemistry

Prior to immunostaining, sections were deparaffinized, hydrated in graded alcohol, and microwave treated for 10 min at 750 W and 15 min at 350 W. Heat-induced epitope retrieval was performed by heating the TMA slides immersed in retrieval buffer pH 6 (Lab Vision, Freemont, CA) for 4 min at 125°C in a pressure boiler (Decloaking chamber, Biocare medical). Immunostaining was performed by avidin-biotin peroxidase staining technique (Vector elite), using 3,3-diaminobenzidine as a substrate. The presence of heterogeneous cell populations in the TMA was documented using the following immunohistochemical markers: GFAP (1∶500; polyclonal rabbit, DAKO, Denmark), vimentin (1∶80; Mouse clone V9, Sigma), neuronal nuclear protein (1∶1000; Mouse clone MAB377, Chemicon, Temecula, CA), microtubule-associated protein (1∶100; Mouse clone HM2, Sigma), CD34 (1∶100; Mouse monoclonal, DAKO), Ki67 (1∶500; Rabbit polyclonal, DAKO), and mutated isocitrate dehydrogenase 1 (IDH1) R132H protein (1∶100; monoclonal mouse antibody, mIDH1R132) [38].

### Fluorescent *in situ* hybridization

All samples included in the TMA were studied by fluorescent *in situ* hybridization analysis (FISH) to identify losses of chromosomal arms 1p and 19q (LOH 1p/19q), as part of our previous study [36]. The commercially purchased probes used for hybridization were Zytolight SPEC 1p36/1q25 and 19q13/19p13 dual color probes (Nordic BioSite, Sweden). Slides were assessed under a fluorescence microscope (Olympus BX 50 Deutschland GmbH), and a minimum of 100 non-overlapping nuclei was calculated for each hybridization. A tumor was considered deleted if >50% of the nuclei harbored two signals of the reference probe but only one signal of the target probe.

### Antibodies for detection of GABA-A channel subunits

Based on the results from the qRT-PCR and the availability of antibodies that could be used for immunostaining of paraffin-embedded formalin fixed tissue, we selected the following antibodies against four different GABA-A channel subunits: One mouse monoclonal antibody against the α1 subunit (GABRA1, CAB22502; Chemicon, Millipore; dilution 1∶50), and three monospecific antibodies, generated through affinity purification of polyclonal antisera, for detection of, respectively: the γ1 subunit (GABRG1, HPA035622; Atlas antibodies, Sigma-Aldrich; dilution 1∶30); the ρ2 subunit (GABRR2, HPA016467; Atlas antibodies, Sigma-Aldrich; dilution 1∶75); the θ subunit (GABRQ, HPA002063; Atlas antibodies, Sigma-Aldrich; dilution: 1∶100) [39].

### Evaluation of immunohistochemistry

The neuropathologists in this study (EA, IA) evaluated all imunostainings and identified immunoreactive cell types as well as the cellular distribution of the proteins. For each sample, the entire piece of micro tissue was examined through light microscopy at magnification 20–40×. The percentage of entrapped neurons, identified by immunoreactivity for neuronal nuclear protein, was estimated for all samples in the TMA. Representative areas containing the highest density of immunoreactive cells for the GABA-A channel subunits were used for counting ≥200 tumor cells per section. The fraction of immunoreactive tumor cells labeled with GABA-A subunit antibodies was then calculated and graded as 0–2 (0 = no positive cells; 1 (+) = few (<10%) positive cells; 2 (++) = several (≥10%) positive cells).

### Characterization of the TMA

Clinical characteristics and histological and molecular characterization of the tumors included in the TMA are shown in Table 3. Of the 91 tumors included in the present study, 4 tumors were re-classified as high-grade gliomas (3 oligoastrocytomas grade III, 1 astrocytoma grade III). The remaining tumors consisted of astrocytoma grad II (n = 42), oligodendroglioma grade II (n = 27) or oligoastrocytoma grade II (n = 18). Histological analysis revealed a fraction of 0–5% of all cells to consist of entrapped neurons (mean percentage in all sections 2.7%). Molecular characterization confirmed the presence of mutated IDH1 R132H protein in 76/91 tumor samples (84%) and LOH 1p/19q in 26/91 tumor samples (29%) (Table 3).

### Statistical analysis

Statistical analysis comparing mRNA levels of individual GABA-A channel subunits between different types of gliomas was carried out using SigmaPlot v11 (Systat Software Inc., USA), and assessed by Kruskal-Wallis ANOVA on ranks, with significance level set to p<0.05. The spearman correlation test was used to calculate the coefficients between α1, γ1, ρ2 and θ subunit protein expression and was performed in JMP statistical software, version 5.0.1a (SAS Institute Inc., Cary, North Carolina, USA).

Survival curves were plotted according to the Kaplan-Meier method (product-limit method) and the log-rank probability test (Mantel-Cox) estimated the prognostic value of each specific clinical, histological, molecular or radiological parameter in the univariate analysis. The Cox proportional hazard model was used to calculate the impact of each prognostic factor in the multivariate analysis. Stepwise exclusion of variables was used to achieve a model with as few variables as possible. The level for confounders to be removed from the model when adjusted for variables already in the model (P-to-remove) was set to >0.1. The natural logarithm (ln) of the cumulative hazard plots was used to confirm the assumption of the proportional hazard functions. Statistical calculations were performed in JMP, version 5.0.1a (SAS Institute Inc., Cary, North Carolina, USA).

## Supporting Information

Figure S1 **Detailed presentation of the RT-PCR results for each of the 17 subunits, showing quantitative mRNA levels between astrocytomas (n = 6) and oligodendrogliomas grade II (n = 6).** The normalized mRNA expression of each target gene relative to a reference gene TATA-binding protein (*TBP*) was calculated using the 2^−ΔCt^ method.
(TIF)

Figure S2 **Detailed presentation of the RT-PCR results for each of the 17 subunits, showing quantitative mRNA levels between astrocytomas (n = 5) and oligodendrogliomas grade III (n = 5).** The normalized mRNA expression of each target gene relative to a reference gene TATA-binding protein (*TBP*) was calculated using the 2^−ΔCt^ method.
(TIF)

Figure S3 **Relative mRNA levels for 17 GABAR_A_ subunits in respectively peri-tumoral tissue (n = 5), gliomas grade II (n = 12), gliomas grade III (n = 10), and glioblastomas (GBM) (n = 7).**
(TIF)

Table S1 **Primer sequences of GABA-A channel subunits used for qRT-PCR.**
(DOC)
