# Supplementary figures and images for: GABA-A Channel Subunit Expression in Human Glioma Correlates with Tumor Histology and Clinical Outcome
Source: PLoS One. 2012 May 17;7(5):e37041. doi: 10.1371/journal.pone.0037041 (PMC3355166; doi:10.1371/journal.pone.0037041)

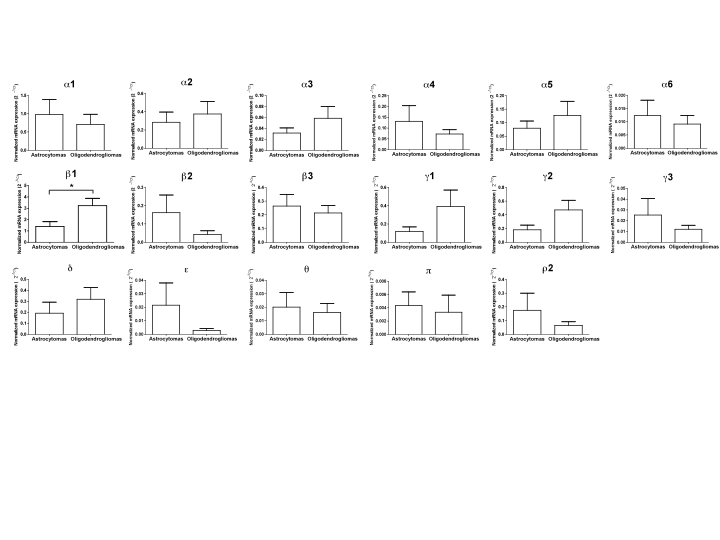

Supplement: Figure S1 — Detailed presentation of the RT-PCR results for each of the 17 subunits, showing quantitative mRNA levels between astrocytomas (n = 6) and oligodendrogliomas grade II (n = 6). The normalized mRNA expression of each target gene relative to a reference gene TATA-binding protein (TBP) was calculated using the 2−ΔCt method. (TIF) [file pone.0037041.s001.tif]

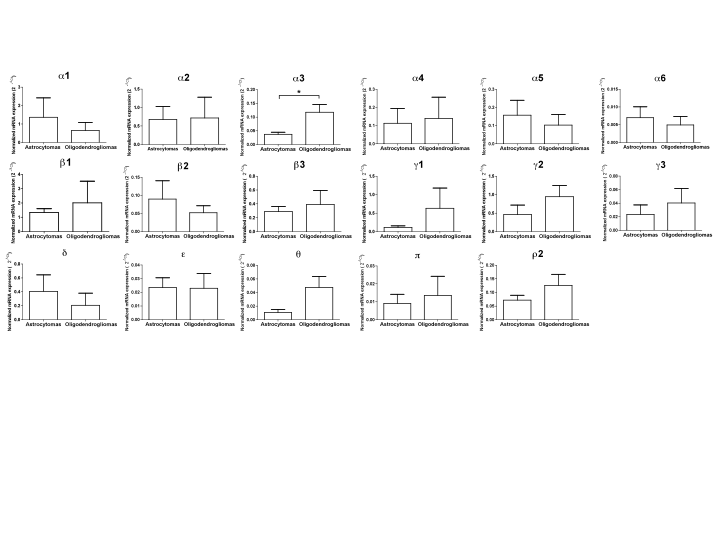

Supplement: Figure S2 — Detailed presentation of the RT-PCR results for each of the 17 subunits, showing quantitative mRNA levels between astrocytomas (n = 5) and oligodendrogliomas grade III (n = 5). The normalized mRNA expression of each target gene relative to a reference gene TATA-binding protein (TBP) was calculated using the 2−ΔCt method. (TIF) [file pone.0037041.s002.tif]

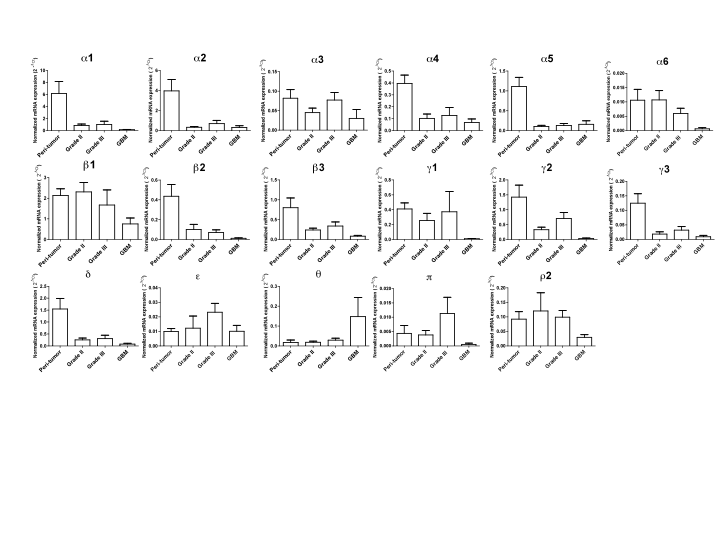

Supplement: Figure S3 — Relative mRNA levels for 17 GABARA subunits in respectively peri-tumoral tissue (n = 5), gliomas grade II (n = 12), gliomas grade III (n = 10), and glioblastomas (GBM) (n = 7). (TIF) [file pone.0037041.s003.tif]
