# Supplementary material for: GABA-A Channel Subunit Expression in Human Glioma Correlates with Tumor Histology and Clinical Outcome
Source: PLoS One. 2012 May 17;7(5):e37041. doi: 10.1371/journal.pone.0037041 (PMC3355166; doi:10.1371/journal.pone.0037041)
Supplement: Table S1 — Primer sequences of GABA-A channel subunits used for qRT-PCR. (DOC) [file pone.0037041.s004.doc]

**Table S1.** Primer sequences of GABA-A channel subunits used for qRT-PCR

| Gene | Primer | Product size (bp) | Reference Number |
| --- | --- | --- | --- |
| α1 (*GABRA1*) | F: GGATTGGGAGAGCGTGTAACC | 66 | NM_000806 |
|  | R: TGAAACGGGTCCGAAACTG |  |  |
| α2 (*GABRA2*) | F: GTTCAAGCTGAATGCCCAAT | 160 | NM_000807 |
|  | R: ACCTAGAGCCATCAGGAGCA |  |  |
| α3 (*GABRA3*) | F: CAACTTGTTTCAGTTCATTCATCCTT | 102 | NM_000808 |
|  | R: CTTGTTTGTGTGATTATCATCTTCTTAGG |  |  |
| α4 (*GABRA4*) | F: TTGGGGGTCCTGTTACAGAAG | 105 | NM_000809 |
|  | R: TCTGCCTGAAGAACACATCCA |  |  |
| α5 (*GABRA5*) | F: CTTCTCGGCGCTGATAGAGT | 105 | NM_000810 |
|  | R: CGCTTTTTCTTGATCTTGGC |  |  |
| α6 (*GABRA6*) | F: ACCCACAGTGACAATATCAAAAGC | 67 | NM_000811 |
|  | R: GGAGTCAGGATGCAAAACAATCT |  |  |
| β1 (*GABRB1*) | F: GTACAAAATCGAGAGAGTCTGGG | 144 | NM_000812 |
|  | R: GCG AAT GTC ATA TCC TTT GAG CA |  |  |
| β2 (*GABRB2*) | F: GCAGAGTGTCAATGACCCTAGT | 137 | NM_021911 |
|  | R: TGGCAATGTCAATGTTCATCCC |  |  |
| β3 (*GABRB3*) | F: CAAGCTGTTGAAAGGCTACGA | 108 | NM_000814 |
|  | R: ACTTCGGAAACCATGTCGATG |  |  |
| γ1 (*GABRG1*) | F: CCTTTTCTTCTGCGGAGTCAA | 91 | NM_173536 |
|  | R: CATCTGCCTTATCAACACAGTTTCC |  |  |
| γ2 (*GABRG2*) | F: CACAGAAAATGACGGTGTGG | 136 | NM_000816 |
|  | R: TCACCCTCAGGAACTTTTGG |  |  |
| γ3 (*GABRG3*) | F: AACCAACCACCACGAAGAAGA | 113 | NM_033223 |
|  | R: CCTCATGTCCAGGAGGGAAT |  |  |
| δ (*GABRD*) | F: ACCACGGAGCTGATGAACTT | 109 | NM_000815 |
|  | R: AGGGCATGTAGGATTGGATG |  |  |
| ε (*GABRE*) | F: TGGATTCTCACTCTTGCCCTCTA | 107 | NM_004961 |
|  | R: GGAGTTCTTCTCATTGATTTCAAGCT |  |  |
| θ (*GABRQ*) | F: CCAGGGTGACAATTGGCTTAA | 63 | NM_018558 |
|  | R: CCCGCAGATGTGAGTCGAT |  |  |
| π (*GABRP*) | F: GGCCTTGCTAGAATATGCAGTTG | 76 | NM_014211 |
|  | R: CTTTGTTGTCCCCCTATCTTTGG |  |  |
| Ρ1 (*GABRR1*) | Hs00266687_m1 from AppliedBiosystem | 94 | NM_002042 |
| ρ2 (*GABRR2*) | F: CCTAGAAGAGGGCATAGACATCG | 99 | NM_002043 |
|  | R: TCCAGTAGCTGCTGCATTGTTTG |  |  |
| ρ3 (*GABRR3*) | F: TGATGCTTTCATGGGTTTCA | 111 | NM_001105580 |
|  | R: CGCTCACAGCAGTGATGATT |  |  |
| *TBP* | F:GAGCTGTGATGTGAAGTTTCC | 117 | NM_003194.3 |
|  | R:TCTGGGTTTGATCATTCTGTAG |  |  |
